# Porcine promyelocytic leukemia protein isoforms suppress Japanese encephalitis virus replication in PK15 cells

**DOI:** 10.1186/s12985-023-02212-x

**Published:** 2023-11-29

**Authors:** Zhenyu Chen, Huaijin Liu, Jingjing Zhu, Xing Duan, Han Wang, Xiangchen Li, Xiaolong Zhou, Ayong Zhao, Songbai Yang

**Affiliations:** Key Laboratory of Applied Technology on Green-Eco-Healthy Animal Husbandry of Zhejiang Province, College of Animal Science and Technology, College of Veterinary Medicine, Zhejiang A&F University, Hangzhou, China

**Keywords:** Porcine, PML isoforms, JEV, IFN-β, IRF3

## Abstract

**Background:**

Promyelocytic leukemia protein (PML) is a primary component of PML nuclear bodies (PML-NBs). PML and PML-NBs play critical roles in processes like the cell cycle, DNA damage repair, apoptosis, and the antiviral immune response. Previously, we identified five porcine PML alternative splicing variants and observed an increase in the expression of these PML isoforms following Japanese encephalitis virus (JEV) infection. In this study, we examined the functional roles of these PML isoforms in JEV infection.

**Methods:**

PML isoforms were either knocked down or overexpressed in PK15 cells, after which they were infected with JEV. Subsequently, we analyzed the gene expression of PML isoforms, JEV, and the interferon (IFN)-β signaling pathway using quantitative reverse transcription-polymerase chain reaction (qRT-PCR) and Western blot. Viral titers were determined through 50% tissue culture infectious dose (TCID_50_) assays.

**Results:**

Our results demonstrated that the knockdown of endogenous PML promoted JEV replication, while the overexpression of PML isoforms 1, 3, 4, and 5 (PML1, PML3, PML4, and PML5) inhibited JEV replication. Further investigation revealed that PML1, PML3, PML4, and PML5 negatively regulated the expression of genes involved in the interferon (IFN)-β signaling pathway by inhibiting IFN regulatory factor 3 (IRF3) post-JEV infection.

**Conclusions:**

These findings demonstrate that porcine PML isoforms PML1, PML3, PML4, and PML5 negatively regulate IFN-β and suppress viral replication during JEV infection. The results of this study provide insight into the functional roles of porcine PML isoforms in JEV infection and the regulation of the innate immune response.

**Supplementary Information:**

The online version contains supplementary material available at 10.1186/s12985-023-02212-x.

## Introduction

Promyelocytic leukemia protein (PML), a member of the tripartite motif-containing (TRIM) family (TRIM19), comprises really interesting new gene (RING), B1/B2 box, and coiled-coil (CC) subdomains [1]. The PML gene, initially identified and named in acute promyelocytic leukemia (APL), is recognized for its t(15;17) translocation, which results in the production of a PML/Retinoic acid receptor alpha (RARA) fusion protein responsible for causing APL [2, 3]. PML, a crucial component of nuclear bodies (NBs), organizes and recruits multiple proteins, including death associated protein (DAXX), speckled protein 100 (SP100), and small ubiquitin modifier 1–3 (SUMO1-3), to form NBs [4]. PML-NBs, which typically range from 0.1 to 1 μm in diameter, manifest as punctate structures within the nucleus [5]. These PML-NBs are involved in a variety of biological processes, such as senescence, tumorigenesis, DNA repair, and antiviral defense.

The interplay between PML and PML-NBs with virus infection is complex. PML and PML-NBs play inhibitory roles in the replication of many viruses. Viral proteins disrupt PML-NBs by degrading NB components that resist antiviral activity [6]. The roles of PML-NBs in DNA viruses, such as those causing herpes infection, have been extensively characterized due to their presence in the cell nucleus [6]. For instance, herpes simplex virus 1 (HSV-1) injects viral DNA into the cell nucleus, and PML-NBs suppress viral gene transcription through epigenetic modification to inhibit the early viral life cycle [6, 7]. However, HSV-1 infected cell protein 0 (ICP0) can disrupt PML-NBs by degrading components such as PML, thereby overcoming the antiviral response [8, 9]. Numerous studies have shown that PML-NBs also regulate RNA virus infection. For instance, PML knockdown enhances human immunodeficiency virus infection in fibroblast cells, but not in T cells or myeloid cell lines, highlighting the cell type-dependent functional roles of PML in virus infection [10]. Additionally, although PML inhibits enterovirus 71 infection by reducing autophagy [11], the viral 3 C protease can disrupt PML-NBs by cleaving PML protein to counteract antiviral activity [11, 12].

A single PML gene can produce several isoforms through alternative splicing. Currently, seven human PML isoforms have been identified, all of which share a common N-terminal domain containing RBCC/TRIM motifs but differ in the C-terminal domain [5]. PML isoforms play distinct roles in various biological processes, including their response to viral infections. Human PML3 and PML4 confer resistance to infection by several viruses. For instance, PML3 and PML4 suppress vesicular stomatitis virus (VSV) replication, and PML4 promotes interferon (IFN)-β production by activating IFN regulatory factor 3 (IRF3) during VSV infection [13]. Among human PML isoforms, only PML3 and PML4 inhibit dengue virus (DENV) infection. Likewise, the DENV non-structural 5 (NS5) protein disassembles PML-NBs by forming complexes with PML3 and PML4 [14, 15]. PML4 inhibits encephalomyocarditis virus replication and protein synthesis by sequestering the three-dimensional viral polymerase [16]. Among the porcine PML isoforms, only PML2 has been demonstrated to suppress pseudorabies virus infection [17].

Japanese encephalitis virus (JEV) is a positive-sense, single-stranded RNA virus that encodes a single, long peptide. This peptide is cleaved into ten proteins, comprising three structural and seven nonstructural proteins [18, 19]. JEV belongs to the *Flaviviridae* family and is responsible for causing acute encephalitis in humans, with transmission occurring through mosquitoes. Annually, approximately 70,000 cases emerge, resulting in 10,000–15,000 deaths, primarily in Asian and Western Pacific countries [20]. Pigs, considered amplifying hosts of JEV, are recognized by epidemiologists as a risk factor for JEV transmission [21]. JEV infection in pigs results in infectious reproductive failures, including stillbirths and fetal abortions, leading to significant economic losses in the pig industry [22, 23].

We previously identified seven porcine PML alternative splicing variants, collectively encoding five distinct proteins. We observed an increase in the expression levels of these PML isoforms following JEV infection [24]. Nevertheless, the roles of these porcine PML isoforms in JEV infection and the immune response remain unclear. In this study, we overexpressed the five porcine PML isoforms in the porcine kidney epithelial cell line 15 (PK15) cells, which were subsequently infected with JEV. The results of this study will offer new insights into the involvement of PML isoforms in the regulation of viral replication and the immune response following JEV infection.

## Materials and methods

### Cell culture

PK15 and baby hamster kidney cell line 21 (BHK-21) cells were obtained from the China Center for Type Culture Collection (CCTCC, Wuhan, China). These cells were cultured in Minimal Essential Medium (MEM; HyClone, Logan, UT, USA) supplemented with 10% fetal bovine serum (FBS; HyClone) and 1% non-essential amino acids (Gibco-BRL Life Technologies, Grand Island, NY, USA). The cultures were maintained in an incubator at 37 °C with 5% CO_2_.

### Antibodies and reagents

Rabbit anti-PML polyclonal antibody was obtained from GeneCreate Biological Engineering Company (Wuhan, China). Rabbit anti-NS3 (GTX125868), NS1 (GTX633820), and envelope (E; GTX125867) polyclonal antibodies were obtained from GeneTex (Irvine, CA, USA). Phospho (p)-nuclear factor kappa B (NF-κB) p65 (Ser536) (#3033) and p-IRF3 (Ser396) (#4947) were purchased from Cell Signaling Technology (Beverly, MA, USA). Rabbit anti-IRF3 (11312-1-AP), NF-κB p65 (10745-1-AP), retinoic acid inducible 1 (RIG-1; 20566-1-AP), mitochondrial antiviral signaling protein (MAVS; 14341-1-AP), and β-actin (20536-1-AP) polyclonal antibodies were purchased from Proteintech (Wuhan, China). Horseradish peroxidase (HRP)-conjugated goat anti-rabbit secondary antibody (A21020) was obtained from Abbkine (Wuhan, China). Recombinant human IFN-β (300-02BC) was obtained from Peprotech (Rocky Hill, NJ, USA).

### Plasmid construction

Overexpression vectors for the five isoforms (pEGFP-C1-PML1/2, PML3, PML4/5, PML6, and PML7) were constructed as described in our previous study [24]. Recombinant pEGFP-C1 plasmids of the five PML isoforms were digested with restriction endonucleases HindIII and BamHI (New England Biolab, MA, USA). Then, the five digested fragments were inserted into the pcDNA3.1 vector (Invitrogen, Carlsbad, CA, USA). These recombinant eukaryotic plasmids were named pcDNA3.1-PML1, pcDNA3.1-PML2, pcDNA3.1-PML3, pcDNA3.1-PML4, and pcDNA3.1-PML5.

### Transfection and small interfering RNA (siRNA) knockdown

siRNA against the common N- terminal sequence of porcine PML was purchased from GenePharma (Shanghai, China), including siPML (GCAAAGAACCAGCCAACUATT), siPML1 (GCUGGUGACUGCACAUCAUTT), and scrambled siRNA (UUCUCCGAACGUGUCACGUTT) sequences. One day prior to transfection, PK15 cells were seeded into 6-well plates for total protein extraction and 12-well plates for RNA extraction. Upon reaching approximately 80% confluency, the cells were transfected with the respective recombinant plasmids or siRNA using the Lipofectamine 3000 transfection reagent (Invitrogen), following the manufacturer’s instructions. At specified time points after transfection, cells were either harvested or exposed to JEV infection for predetermined durations.

### Virus and infection

The JEV strain SA14-14-2 was employed in this study. PK15 cells were infected with JEV (multiplicity of infection = 1) diluted in MEM. After the virus adsorbed to the cells for 1 h, the cells were washed three times with phosphate-buffered saline (PBS). The cells were maintained in MEM with 2% FBS. The infected cells and supernatant were collected at 36 h post-infection (hpi), except for indicated time points after infection. Viral propagation was conducted in BHK-21 cells. Viral titers were determined in BHK-21 cells using a 50% tissue culture infectious dose (TCID_50_) assay and analyzed according to the Reed-Muench method.

### Quantitative reverse transcription-polymerase chain reaction (qRT-PCR) gene expression analysis

To examine mRNA expression levels, PK15 cells were collected, and their total RNA was extracted using TRIzol reagent (Invitrogen) following the manufacturer’s instructions. A total of 1 µg RNA was used to synthesize cDNA with an RT-PCR reagent kit (CoWin Biosciences, Beijing, China). We conducted qRT-PCR analysis using SYBR Premix Ex Taq II (Takara, Dalian, China) on a CFX96 Real-Time System (Bio-Rad, Hercules, CA, USA). The qPCR primer sequences are listed in Table 1. The qPCR amplification conditions were as follows: 95 °C for 2 min, followed by 40 cycles of 95 °C for 5 s, 60 °C for 30 s, and 72 °C for 20 s, ending with a melting curve analysis. Relative gene expression levels were calculated using the 2^−ΔΔCt^ method, with GAPDH serving as the reference gene [25].

**Table 1:** The primer sequences used for qRT-PCR

Names	Primer sequence (5′~3′)	Size (bp)
PML	F: CGGAAGGAAGCCAAATGC	136
	R: TATCCAGGGCCTGCGTGT	
PML1	F: CCACAAGAGGGCCTGAAGAA	109
	R: TGTCGAAGTAGGTGCCCAGA	
PML2,3,4	F: CCTCTGGGCCTCTGCCGGGATG	60
	R: GGCCTGGGAGCAGCAGAGTCCTTGC	
PML5	F: GGACAGGAAGCTCGCTCAT	127
	R: CAGGCAAGCACCCAACAT	
JEV-E	F: GTCCATAGGGAGTGGTTTCA	257
	R: CCTTTCAGAGCCAGTTTGTC	
IFN-β	F: CGATACCAACAAAGGAGCAG	228
	R: GGTTTCATTCCAGCCAGT	
IFN-α	F: GATCAGCAGCTCAGGGAC	208
	R: GCAGGTTTGTGGAGGAAG	
IL-6	F: AGATGCCAAAGGTGATGC	226
	R: CTCCTGATTGAACCCAGA	
TNF-α	F: GCCTCAGCCTCTTCTCCTT	235
	R: GCATTGGCATACCCACTCT	
ISG15	F: TGATGGCATCGGACCTGA	224
	R: GACCTCATAGGCGTTGCTG	
MX1	F: ATCACCAGGGTAGCTGTAGG	232
	R: TGTCCTCAGTGCCTTTGTC	
RIG-1	F: GCCACAACACCAGCAAAC	116
	R: AACCGAGGCAGTCAGTCC	
GAPDH	F: GGACTCATGACCACGGTCCAT	220
	R: TCAGATCCACAACCGACACGT	

### Western blot analysis

Total protein was isolated from PK15 cells using radioimmunoprecipitation assay buffer, which was supplemented with phosphatase and protease inhibitor cocktails (CoWin Biosciences). Equal amounts of total protein were separated using sodium dodecyl sulfate–polyacrylamide gel electrophoresis and transferred to polyvinylidene difluoride (PVDF) membranes (Millipore, Billerica, MA, USA). The PVDF membranes were blocked with 5% nonfat milk diluted in Tris-buffered saline containing 0.1% Tween-20 (TBST) for 1 h. Following incubation with primary antibodies overnight at 4 °C, the membranes were washed three times with TBST and then incubated with the corresponding HRP-conjugated secondary antibodies at room temperature for 1 h. Finally, the membranes were treated with Immobilon Western Chemiluminescent HRP Substrate (Merck Millipore, Darmstadt, Germany). β-actin was used as an internal control. The targeted protein bands were imaged using a Tanon 5200 system (Tanon Science and Technology, Shanghai, China). Band density was analyzed using the ImageJ software (National Institutes of Health, Bethesda, MD, USA).

### Cell viability assays

Cell viability was measured using a Cell Counting Kit-8 (CCK-8) assay kit (Beyotime Biotechnology, Shanghai, China). Briefly, PK15 cells were plated on a 96-well culture plate 1 day before transfection. The cells were transfected with one of the five isoform recombinant plasmids or empty vector, washed three times with PBS at 36 h post-transfection, and then incubated with 10 µL of CCK-8 reagent diluted in 90 µL of fresh MEM for 4 h at 37 °C. Cell viability was determined by detecting absorbance at a wavelength of 450 nm using an iMARK microplate reader (Bio-Rad).

### Statistical analyses

Data are presented as means ± standard error of the mean of three (qRT-PCR and TCID_50_) or six (CCK-8 assay) biological replicates, with three (Western blot) independent experiments. Statistical analyses included unpaired, two-tailed Student’s *t*-test (two groups) and one-way analysis of variance followed by Dunnett’s test for multiple groups using the GraphPad Prism software (GraphPad Software, San Diego, CA, USA). Statistical significance was evaluated at *P* < 0.05 (*), *P* < 0.01 (**), *P* < 0.001 (***), and *P* < 0.0001 (****).

## Results

### Porcine PML knockdown enhances JEV replication

**Fig. 1:**
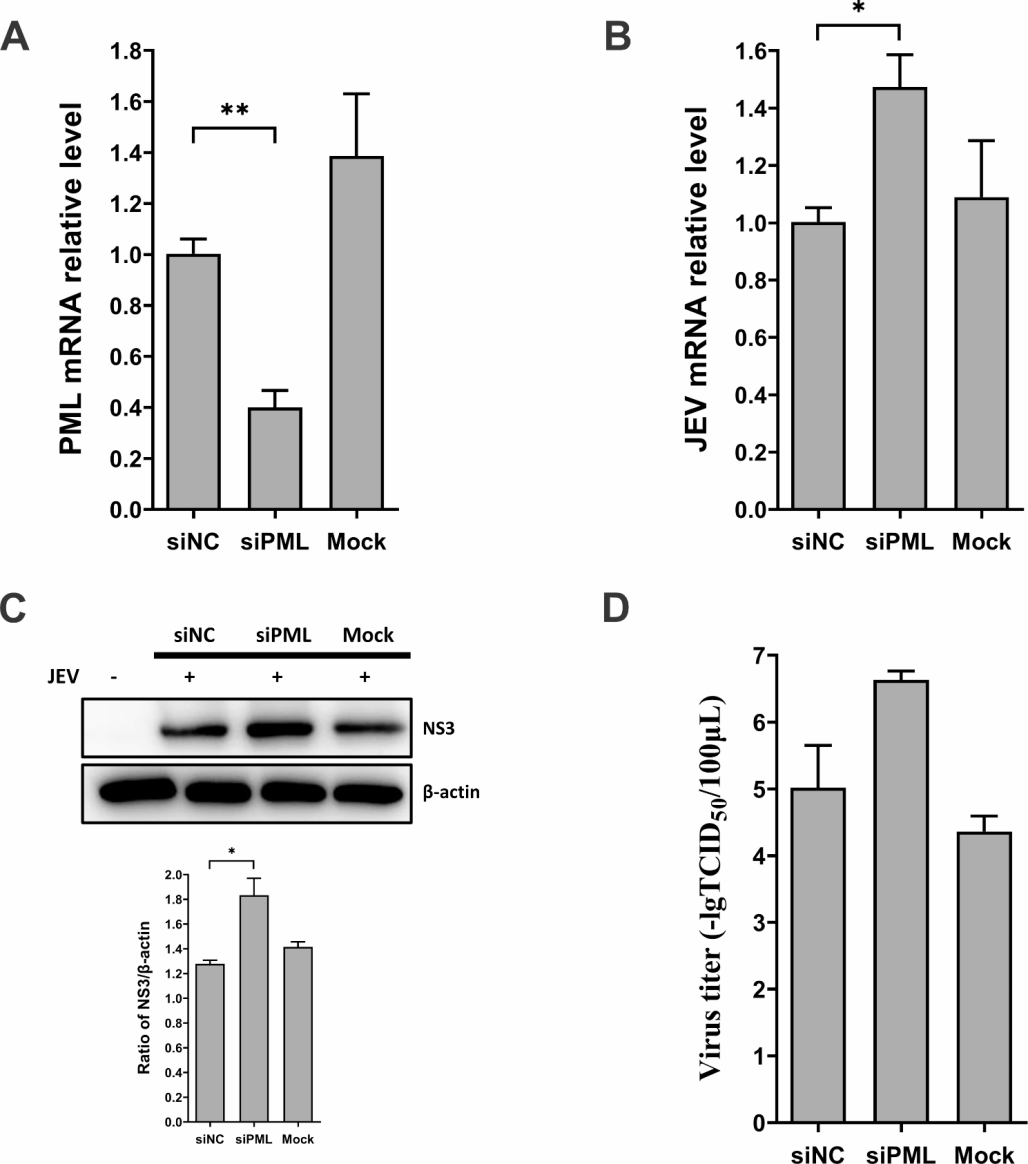
Promyelocytic leukemia protein (PML) expression knockdown promotes Japanese encephalitis virus (JEV) infection. (**A**) PK15 cells were transfected with either porcine PML-specific siRNA (siPML) or non-targeting negative control siRNA (siNC) for 36 h. Subsequently, we assessed PML mRNA expression using qRT-PCR. Following a 36 h transfection with siPML or siNC, PK15 cells were infected with JEV for an additional 36 h. We assessed (**B**) JEV mRNA expression, (**C**) NS3 protein levels, and (**D**) viral titer using qRT-PCR, Western blot analysis, and TCID_50_ assays, respectively

PML is an important host restriction factor for multiple viral infections. To explore the role of porcine PML in JEV infection, we designed siRNA specifically targeting the common N-terminal sequence shared among all porcine alternative isoforms. The siRNA was transfected into PK15 cells, which were then infected with JEV. PML expression levels were significantly reduced after siRNA transfection compared to the control group (Fig. 1A). JEV mRNA levels exhibited a significant increase in PML knockdown cells (Fig. 1B). Western blot analysis revealed an elevation in JEV protein levels following PML knockdown (Fig. 1C). Although there was an observed rise in viral titer in PML-siRNA transfected cells compared to the scrambled-siRNA group, the difference did not reach statistical significance (Fig. 1D). In a similar manner, PK15 cells were transfected with specific siRNA targeting the PML1 isoform, followed by JEV infection. The results indicated significant inhibition in the expression levels of the PML1 isoform and total PML, while other isoforms remained unaffected (Fig. S1). Interestingly, the mRNA levels of both JEV and IFN-β exhibited a significant increase following PML1 isoform knockdown compared to the scrambled-siRNA group (Fig. S1). These results highlight that JEV infection is enhanced after the knockdown of the porcine PML1 isoform.

### Porcine isoforms PML1, PML3, PML4, and PML5 inhibit JEV replication

**Fig. 2:**
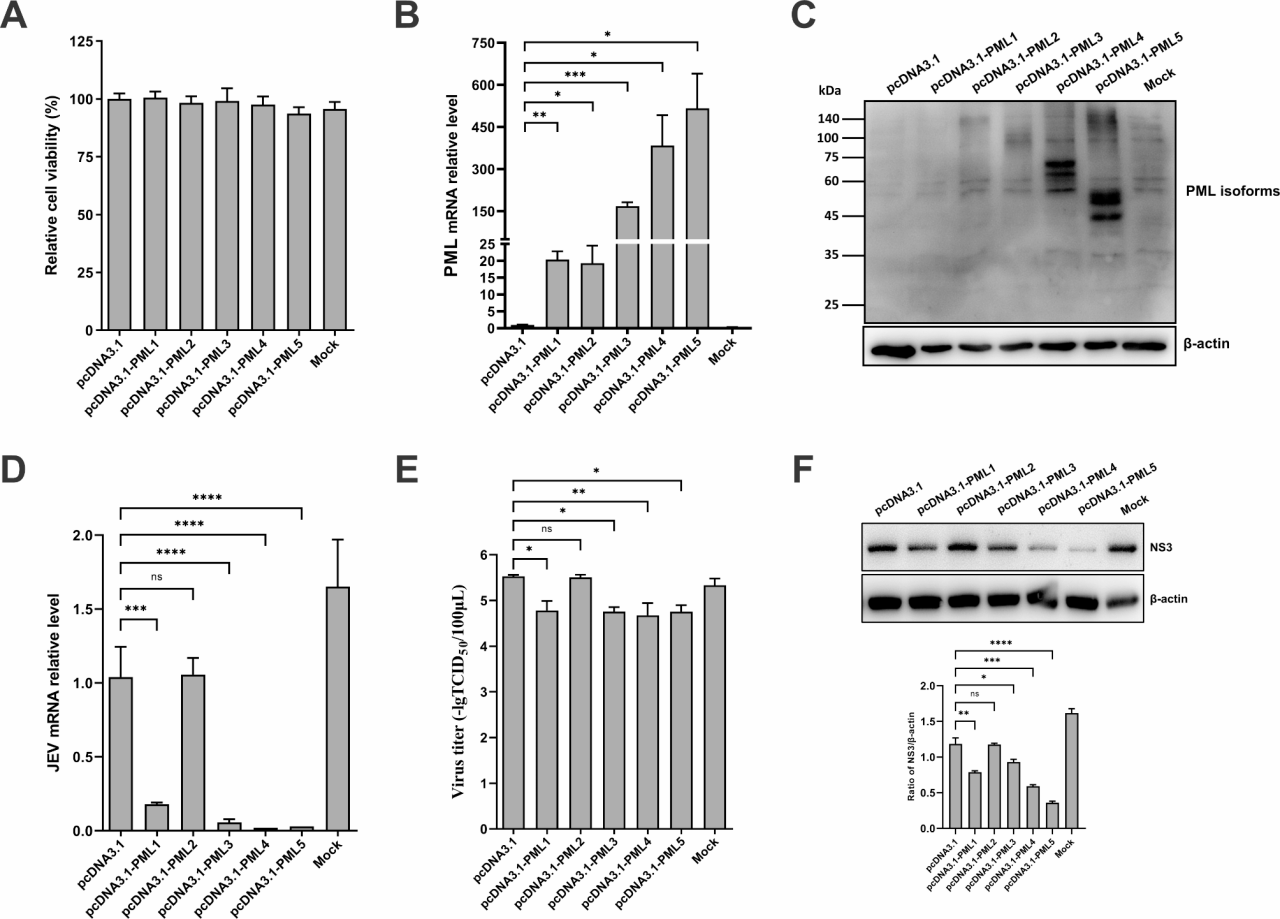
Effects of porcine PML isoform overexpression on JEV infection. PK15 cells were transfected with recombinant plasmids encoding five PML isoforms or an empty vector plasmid (control group) for 36 h. Subsequently, we assessed (**A**) cell viability and the (**B**) mRNA and (**C**) protein expression of the PML isoforms. PK15 cells were transfected with plasmid DNA for 36 h, and then infected with JEV for an additional 36 h. We assessed the (**D**) mRNA expression, (**E**) viral titer, and (**F**) protein levels of JEV

We had previously identified five alternative splicing variants of porcine PML, which were found to be significantly upregulated in JEV-infected cells [24]. To explore the functional roles of these PML isoforms in JEV infection, PK15 cells were transfected with recombinant plasmids corresponding to one of the five isoforms or an empty vector. At 36 h post-transfection, cells were harvested for RNA and protein isolation. The results indicated that plasmid transfection did not have a significant impact on cell viability (Fig. 2A). The qRT-PCR results demonstrated a significant increase in the mRNA expression levels of all five PML isoforms after transfection, in comparison to the empty vector group (Fig. 2B). Since we did not have access to a commercially available anti-PML antibody for pigs, we customized a rabbit polyclonal anti-PML antibody using the common N-terminal amino acid sequence shared by all PML isoforms. This antibody recognized isoforms PML2-5. However, Western blot analysis did not reveal a specific PML1 protein band, suggesting that PML1 is not recognized by this antibody. Western blot analysis clearly indicated increased levels of PML2, PML3, PML4, and PML5 proteins after transfection when compared with the control group (Fig. 2C).

To assess JEV replication after overexpression of the porcine isoforms, cells were infected with JEV for 36 h after transfection. JEV replication was examined using qRT-PCR, Western blot, and TCID_50_ analyses. The results indicated that the levels of JEV mRNA (Fig. 2D), viral titer (Fig. 2E), and JEV-NS3 protein (Fig. 2F) were significantly suppressed in PML1, PML3, PML4, and PML5 overexpressing cells compared to the empty vector control group. These results suggest that the overexpression of PML1, PML3, PML4, and PML5 isoforms can inhibit JEV infection in PK15 cells.

**Porcine isoforms PML1, PML3, PML4, and PML5 negatively regulate the IFN-β signaling pathway after JEV infection**.

**Fig. 3:**
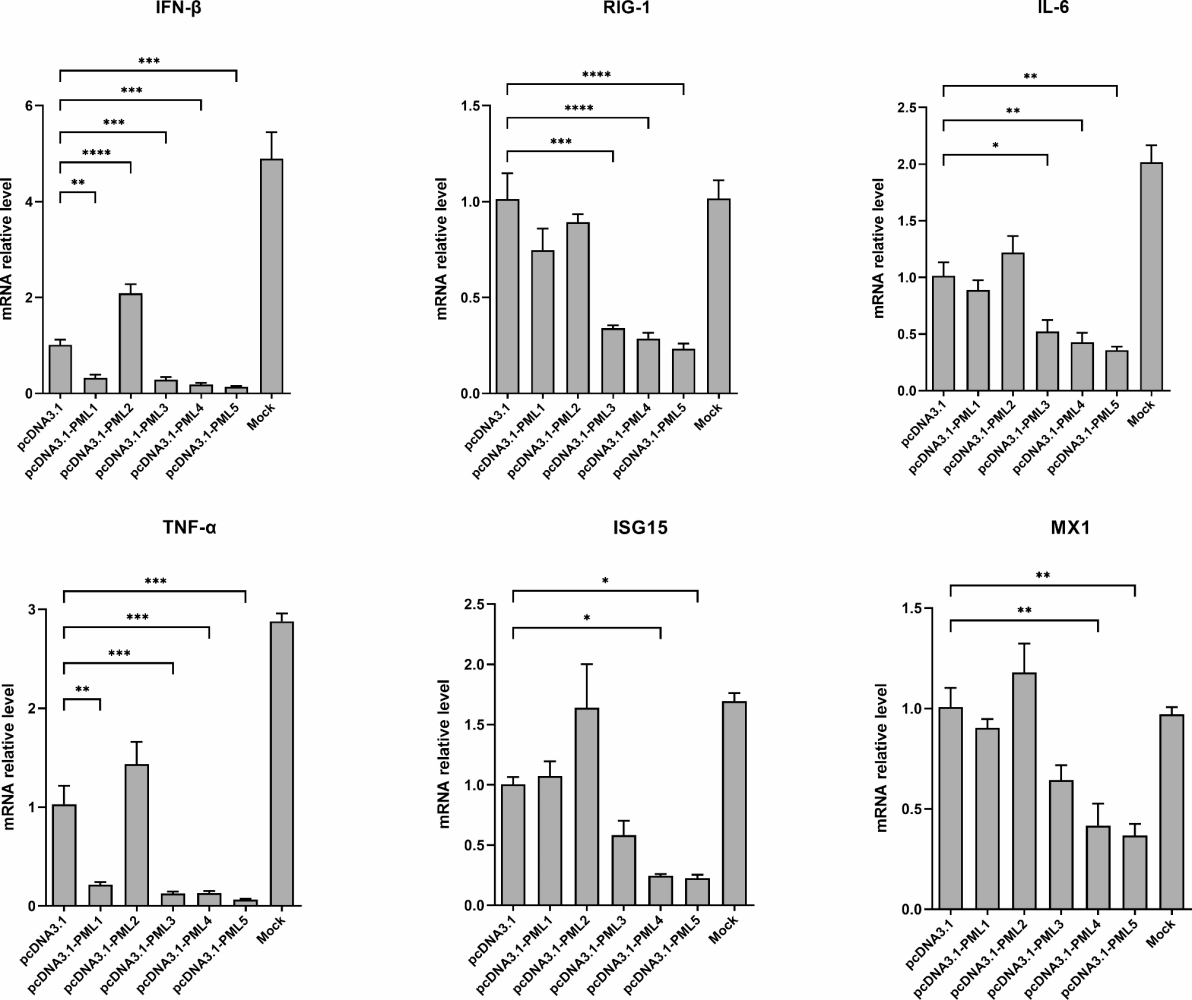
mRNA expression of interferon (IFN)-β signaling in PML isoform overexpressing cells during JEV infection. PK15 cells were transfected with recombinant plasmids of five PML isoforms or empty vector plasmid (control group) for 36 h, and then infected with JEV for an additional 36 h. The mRNA expression of IFN-β signaling and pro-inflammatory cytokines was detected using qRT-PCR, and relative expression levels are expressed as fold changes relative to the control group

PML plays a crucial role as a coregulator in antiviral innate immunity [6, 13]. To investigate whether these porcine PML isoforms are involved in the innate immune response, we transfected overexpression plasmids of the five PML isoforms into PK15 cells. At 36 h post-transfection, the cells were collected for qRT-PCR analysis or infected with JEV for an additional 36 h. We examined the mRNA expression of genes involved in the innate immune response, including IFNs, the IFN-stimulated genes ISG15 and MX1, as well as the pro-inflammatory cytokines interleukin (IL)-6 and tumor necrosis factor (TNF)-α, using qRT-PCR analysis. The results indicated that the mRNA expression levels of IFN-β, RIG-1, IL-6, ISG15, and MX1 were elevated in cells overexpressing PML1, PML3, PML4, and PML5 compared to the control cells (Fig. S2). However, the mRNA expression levels of IFN-β and TNF-α were significantly reduced in the PML1, PML3, PML4, and PML5 overexpression groups compared to the control group after JEV infection. Additionally, the mRNA expression levels of RIG-1, IL-6, ISG15, and MX1 decreased in PML3, PML4, and PML5 overexpressing cells compared to control cells during JEV infection (Fig. 3). In all PML isoforms overexpressing cells, mRNA expression of IFN-α was not significantly altered during JEV infection (Fig. S3). Remarkably, PML2 significantly enhanced the mRNA expression levels of IFN-β during JEV infection but did not significantly affect the expression levels of other genes related to the innate immune response (Fig. 3). These results suggest that PML1, PML3, PML4, and PML5 positively regulate the IFN-β immune response, while negatively regulating the mRNA expression of IFN-β, ISGs, and pro-inflammatory cytokines following JEV infection.

### Effects of IFN-β treatment on JEV replication

**Fig. 4:**
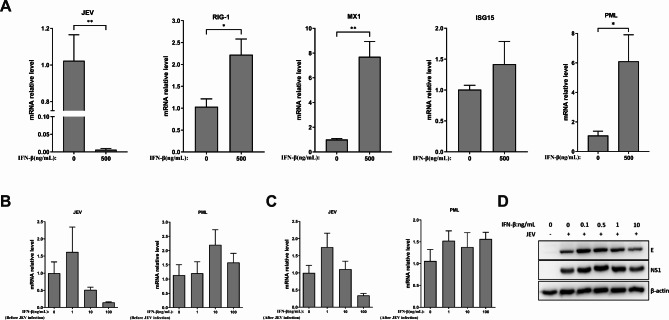
Effects of IFN-β treatment on JEV replication. (**A**) PK15 cells were treated with 500 ng/mL IFN-β for 24 h, and then infected with JEV for 36 h. The mRNA expression levels of JEV, IFN-stimulated gene 15 (ISG15), MX1, retinoic acid inducible 1 (RIG-1), and PML were assessed by qRT-PCR analysis. (**B**) PK15 cells were treated with 0, 1, 10, or 100 ng/mL IFN-β for 24 h, and then infected with JEV for 36 h. The expression of JEV and PML mRNA was evaluated by qRT-PCR. (**C**) PK15 cells were infected with JEV for 36 h in the presence of 0, 1, 10, or 100 ng/mL IFN-β, and then the expression of JEV and PML mRNA was assessed. The untreated group (0 ng/mL) was used as a control group. Relative expression levels were expressed as fold changes relative to the control group. (**D**) PK15 cells were infected with JEV for 36 h in the presence of 0, 0.1, 0.5, 1, or 10 ng/mL IFN-β, and then JEV protein levels were assessed by Western blot analysis

Type I IFN (IFN-I) responses, including the IFN-β response, play a critical role in defending against viral infections. IFN-I induces the expression of hundreds of ISGs that directly inhibit almost every step of the viral life cycle [26]. In this study, we found that porcine PML isoforms PML1, PML3, PML4, and PML5 suppress JEV infection. The mRNA expression of IFN-β was also reduced following PML1, PML3, PML4, and PML5 overexpression during JEV infection. To investigate the roles of IFN-β in JEV infection, PK15 cells were exposed to 500 ng/mL recombinant IFN-β for 24 h, after which they were subjected to JEV infection in the absence of IFN-β in the culture medium. At 36 hpi, we examined the mRNA expression of JEV and ISGs using qRT-PCR. The results demonstrated a significant reduction in the mRNA expression of JEV after IFN-β treatment compared to the untreated group (Fig. 4A). In addition, the expression levels of ISG15, MX1, RIG-1, and PML were upregulated in the IFN-β treatment group (Fig. 4A). Next, we investigated the effects of low concentrations of IFN-β on JEV replication. PK15 cells were exposed to 0, 1, 10, or 100 ng/mL IFN-β either before or after JEV infection. The results indicated that a concentration of 1 ng/mL IFN-β slightly increased JEV mRNA expression, while treatment with 1, 10, or 100 ng/mL of IFN-β did not significantly affect the expression of PML (Fig. 4B, C). Lower concentrations of IFN-β (0.1 or 0.5 ng/mL) enhanced the expression of JEV E and NS1 proteins (Fig. 4D). These findings suggest that high concentrations of exogenous IFN-β suppress viral infection, while lower concentrations appear to promote viral replication.

### Porcine PML1, PML3, PML4, and PML5 inhibit the activation of IRF3, rather than NF-κB, during JEV infection

**Fig. 5:**
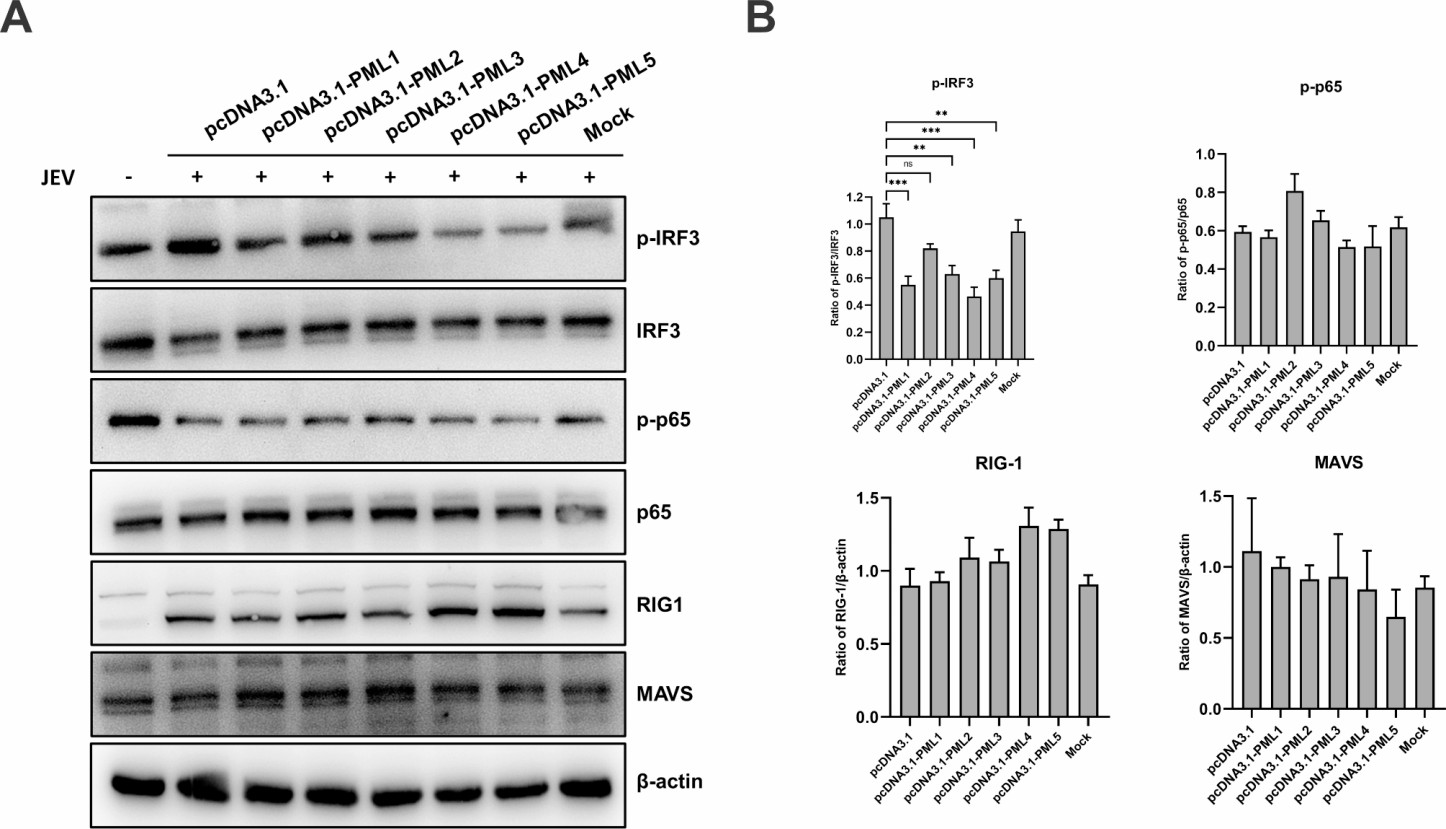
PML1, PML3, PML4, and PML5 suppress IFN regulatory factor 3 (IRF3) but not nuclear factor kappa B (NF-κB) during JEV infection. PK15 cells were transfected with PML isoform overexpression vectors or an empty vector for 36 h, Afterward, they were infected with JEV for an additional 36 h and harvested for protein extraction. (**A**) Protein expression was detected by Western blot analysis. (**B**) Quantitative protein levels in (**A**)

The innate immune system detects pathogens through pattern recognition receptors (PRRs), such as RIG-1. PRRs trigger the activation of either the IRF3 or NF-κB pathways, leading to the transcriptional activation of IFN genes and cytokines [27]. To investigate the pathways involved in IFN-β suppression, we transfected PK15 cells with overexpression plasmids of each of the five porcine PML isoforms, followed by JEV infection. We then detected the protein expression of immune response-related genes using Western blot analysis (Fig. 5). After JEV infection, the PML1, PML3, PML4, and PML5 isoforms significantly inhibited the phosphorylation of IRF3 but had no effect on NF-κB p65. These results indicate that PML1, PML3, PML4, and PML5 suppress JEV-induced p-IRF3 and do not affect the NF-κB pathway.

## Discussion

Numerous studies have demonstrated the role of PML in antiviral responses, limiting infection by multiple viruses [5, 6, 28, 29]. In our previous study, we identified five alternative porcine PML splicing variants that were upregulated during JEV infection [24]. In this study, JEV replication increased after total PML knockdown, suggesting that porcine PML may also have an antiviral role against JEV infection. The overexpression of PML1, PML3, PML4, and PML5 significantly inhibited JEV infection. A recent study demonstrated that human PML1 promoted human adenovirus (HAdV) replication in HepG2 cells, while PML5 and PML6 inhibited it. However, in H1299 cells, PML2 promoted HAdV replication, while PML3, PML4, and PML6 inhibited it [30]. These results highlight that PML isoforms have distinct roles in virus infection, depending on the cell type. The mRNA sequence of porcine PML2 contains a larger fragment of the retained intron 7 sequence at the 3’ end compared to the other four PML isoforms examined in our study [24]. As a result, we speculate that the distinctive protein structure of porcine PML2, characterized by its unique C-terminal domain, may not possess the same antiviral activity as the other PML isoforms.

Host cells recognize pathogens through PRRs, leading to the transcriptional activation of IFNs and the induction of numerous ISGs that exert direct antiviral effects [27, 31]. PML and PML-NBs are involved in the regulation of the innate immune response. Studies have demonstrated that the numbers of NBs and the expression of PML are induced following IFN treatment [6, 32, 33]. Hence, PML plays a pivotal role in the IFN-induced antiviral stage [34]. PML2 has been observed to activate the gene expression of IFN-β and ISGs by recruiting several transcription factors, including NF-κB, signal transducer and activator of transcription 1 (STAT1), coactivator CREB-binding protein (CBP), and IRF3, to form a transcriptional complex [35]. PML4 positively regulates the innate immune response by enhancing IFN-β production [13]. PML activates IFN-γ, but not IFN-α-induced p-STAT1, and ISG expression, indicating that PML positively regulates the IFN-γ signaling pathway [36]. These findings suggest a positive feedback regulation between PML and the innate immune response. Our results demonstrated that the expression levels of IFN-β and TNF-α were significantly reduced in PML1, PML3, PML4, and PML5 overexpression cells during JEV infection. Additionally, the expression levels of RIG-1, IL-6, ISG15, and MX1 were decreased in PML3, PML4, and PML5 overexpression cells during JEV infection, indicating that porcine PML1, PML3, PML4, and PML5 negatively regulate the innate immune response following JEV infection. The overexpression of PML1, PML3, PML4, and PML5 also increased IFN-β and ISG expression in the absence of JEV infection, suggesting that porcine PML isoforms positively regulate the innate immune response. However, JEV can manipulate the PML-mediated innate immune response after infection. Studies have demonstrated that viruses can target various members of the TRIM family to counteract IFN production [37]. For instance, JEV infection upregulated TRIM21 expression, and TRIM21 overexpression inhibited JEV-induced p-IRF3 and IFN-β expression [38]. The Middle East respiratory syndrome (MERS) coronavirus nucleocapsid protein inhibited the production of type I and type III IFNs by sequestering TRIM25 [39]. Therefore, we speculate that JEV may interact with PML1, PML3, PML4, and PML5 to suppress the innate immune response during infection. Further investigation is necessary to elucidate the mechanisms by which JEV suppresses the innate immune response through interaction with PML.

This study presents the first evidence that during JEV infection, both viral infection and innate immune responses are simultaneously suppressed in PML isoform-overexpressing cells. We speculate that this phenomenon may result from two possible mechanisms. First, in the absence of viral infection, PML1, PML3, PML4, and PML5 promote the expression of genes associated with the innate immune response. After JEV infection, the virus suppresses the innate immune response by hijacking the PML isoforms. Moreover, PML isoforms and PML-NBs have the capability to directly inhibit JEV replication, independent of the IFN-β pathway. Furthermore, PML isoforms can shuttle between the nucleus and cytoplasm to suppress viral replication [40]. Another potential mechanism is that IFN-β may facilitate viral replication. Although this mechanism may appear contradictory, studies have shown that IFN can promote porcine circovirus 2 (PCV2) infection. For instance, the knockout of nucleosome assembly protein 1-like 4 (NAP1L4) increased IFN-β, thereby promoting PCV2 replication. Additionally, the addition of exogenous IFN-β in cell culture also facilitated viral replication [41]. The induction of endogenous IFN-β expression in PK15 cells by PCV2 may promote PCV2 replication [42]. In this study, the mRNA expression of IFN-β significantly increased (data not shown) at the late stage of infection, which is consistent with the elevated viral mRNA and protein levels. These findings suggest a positive relationship between IFN-β expression and JEV replication. As a result, we hypothesized that IFN-β may promote JEV replication. To test this hypothesis, we treated PK15 cells with exogenous IFN-β and subsequently infected them with JEV. The findings demonstrated that higher-concentration IFN-β treatment inhibited JEV replication. Nevertheless, JEV replication was increased following treatment with lower IFN-β concentrations (0.1, 0.5, and 1 ng/mL), both before and after JEV infection. These findings suggest that PML isoforms may allow JEV to utilize the innate immune response for its own replication.

Following virus recognition by PRRs, the activation of transcription factors IRF3 and NF-κB is required for IFN-β transcription activation [27]. Previous studies have demonstrated that JEV infection can activate the IRF3 and NF-κB pathways [43, 44]. In this study, the protein expression of p-IRF3 decreased after the overexpression of PML1, PML3, PML4, and PML5 during JEV infection. However, PML1, PML3, PML4, and PML5 did not affect the levels of p-p65 protein after JEV infection. These results suggest that PML1, PML3, PML4, and PML5 inhibited p-IRF3 but did not influence NF-κB activation during JEV infection. In a previous study, it was demonstrated that human PML4 recruited peptidyl-prolyl cis-trans isomerase NIMA-interacting 1 (Pin1) into NBs and inhibited Pin1-induced degradation of IRF3 [13]. Hence, it is plausible that porcine PML isoforms or PML-NBs may interact with negative or positive regulators of p-IRF3, leading to p-IRF3 degradation and subsequently inhibiting IRF3-induced IFN-β production. During RNA virus infection, RIG-1 recognizes the virus and interacts with MAVS, which then activates TANK-binding kinase 1 (TBK1) to induce the phosphorylation of IRF3 [45, 46]. The protein levels of RIG-1 and MAVS did not exhibit significant changes in PML overexpression cells during JEV infection. In contrast, PML3, PML4, and PML5 suppressed RIG-1 mRNA expression following JEV infection. These findings suggest that PML1-mediated IRF3 inhibition may be independent of the RIG-1-MAVS pathway. Nevertheless, further study is needed to elucidate the specific mechanism by which the PML isoforms inhibits p-IRF3 after JEV infection.

## Conclusions

In conclusion, the roles of porcine PML isoforms in JEV replication were identified in the present study, and our results demonstrated that porcine PML1, PML3, PML4, and PML5 suppressed JEV infection. In cells overexpressing PML1, PML3, PML4, and PML5, IRF3 was inactivated to diminish IFN-β transcription during JEV infection. These findings will contribute to a deeper understanding of the regulation of PML-induced viral immune response.

### Electronic supplementary material

Below is the link to the electronic supplementary material.

Additional File 1: Fig. S1 The siRNA targeting the PML1 isoform was transfected into PK15 cells for 24 hours, followed by an additional 36 h of infection with JEV. The mRNA expression levels of the PML1 isoform, total PML, PML2,3,4, PML5, JEV, and IFN-β were then analyzed using qRT-PCR analysis.

Additional File 2: Fig. S2 PK15 cells were transfected with recombinant plasmids of five PML isoforms or empty vector plasmid (control group) for 36 h. The mRNA expression of IFN-β signaling pathway was detected using qRT-PCR. Relative expression levels are expressed as fold changes relative to the control group (see Fig. 3).

Additional File 3: The mRNA expression of IFN-α in overexpressed PML isoform cells during JEV infection (see Fig. 3).

## Data Availability

The datasets used and/or analyzed during the current study are available from the corresponding author on reasonable request.
