# Supplementary figures and images for: Porcine promyelocytic leukemia protein isoforms suppress Japanese encephalitis virus replication in PK15 cells
Source: Virol J. 2023 Nov 29;20:280. doi: 10.1186/s12985-023-02212-x (PMC10687900; doi:10.1186/s12985-023-02212-x)

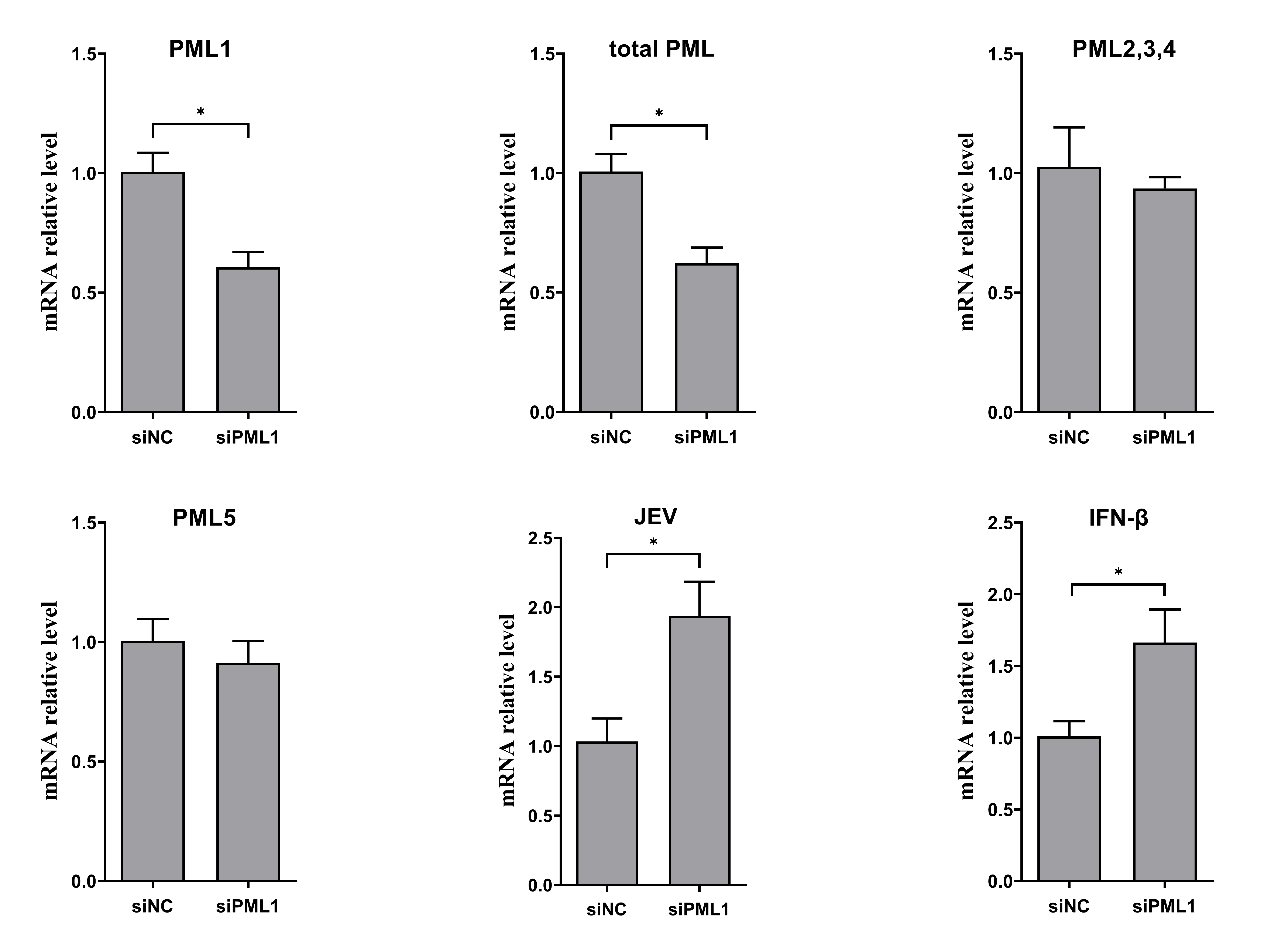

Supplement: Supplementary file 1 — Additional File 1: Fig. S1 The siRNA targeting the PML1 isoform was transfected into PK15 cells for 24 hours, followed by an additional 36 h of infection with JEV. The mRNA expression levels of the PML1 isoform, total PML, PML2,3,4, PML5, JEV, and IFN-β were then analyzed using qRT-PCR analysis. [file 12985_2023_2212_MOESM1_ESM.tif]

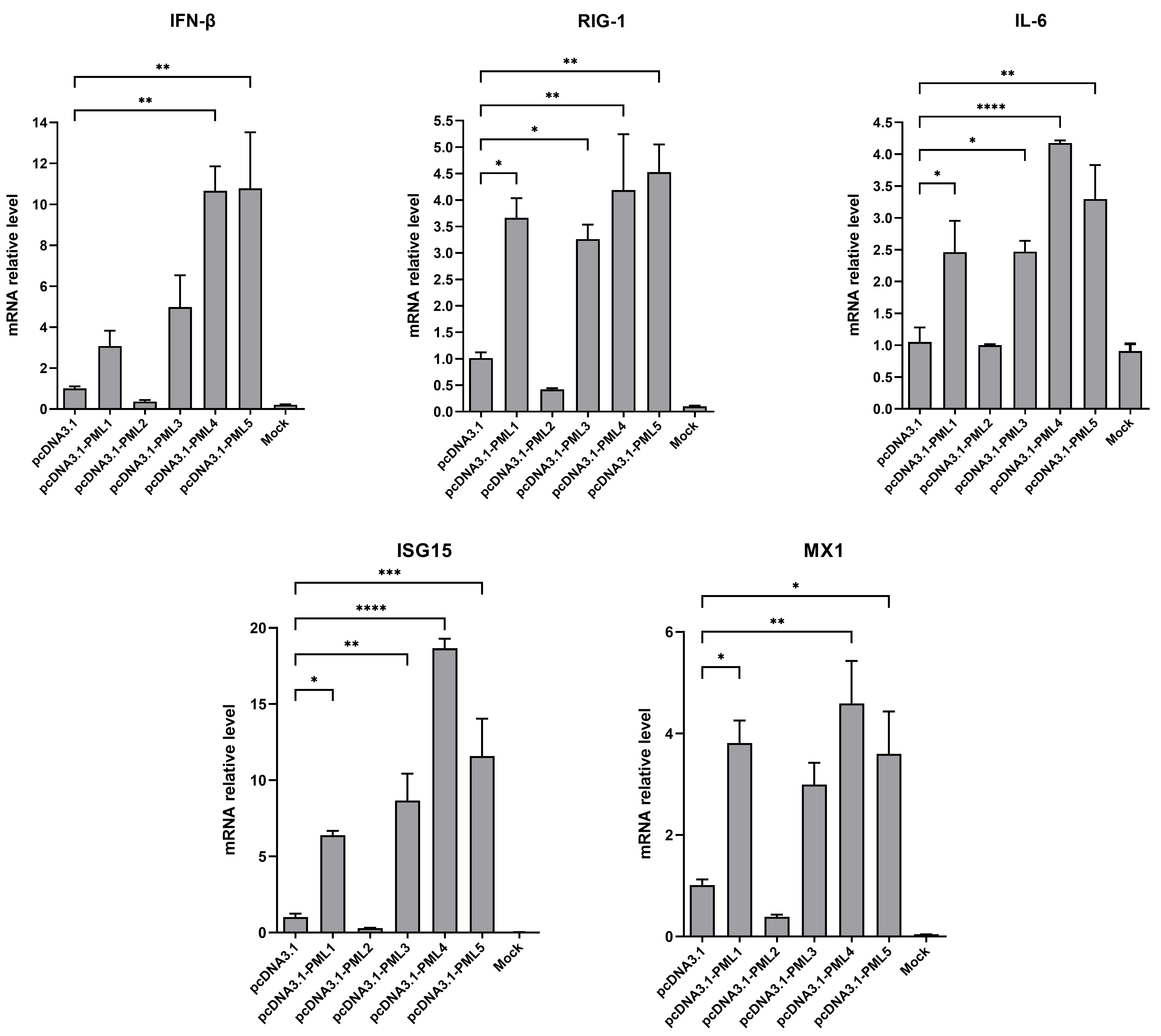

Supplement: Supplementary file 2 — Additional File 2: Fig. S2 PK15 cells were transfected with recombinant plasmids of five PML isoforms or empty vector plasmid (control group) for 36 h. The mRNA expression of IFN-β signaling pathway was detected using qRT-PCR. Relative expression levels are expressed as fold changes relative to the control group (see Fig. 3). [file 12985_2023_2212_MOESM2_ESM.tif]

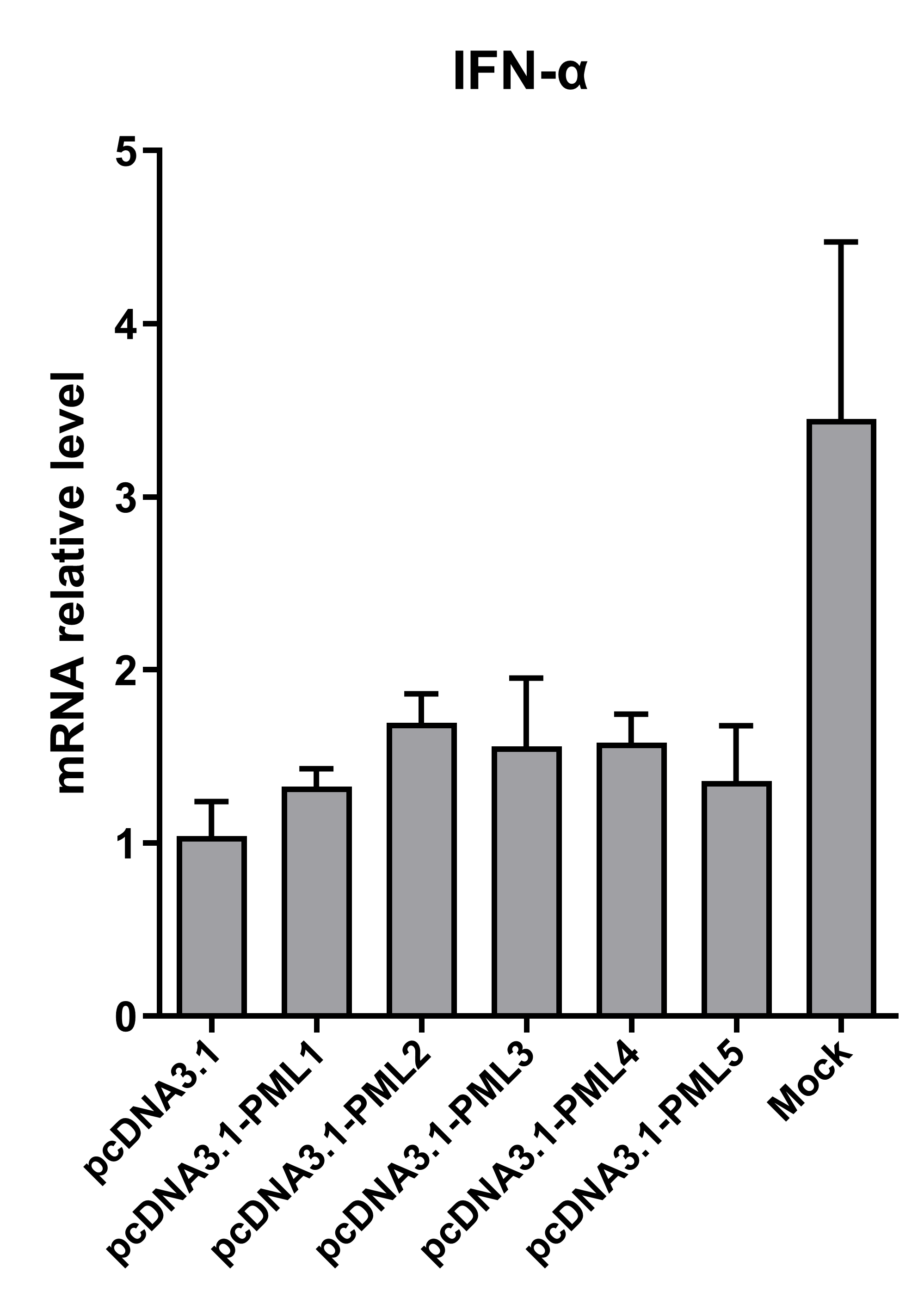

Supplement: Supplementary file 3 — Additional File 3: The mRNA expression of IFN-α in overexpressed PML isoform cells during JEV infection (see Fig. 3). [file 12985_2023_2212_MOESM3_ESM.tif]
